# Supplementary material for: Diagnostic Validation of a Comprehensive Targeted Panel for Broad Mutational and Biomarker Analysis in Solid Tumors
Source: Cancers (Basel). 2022 May 16;14(10):2457. doi: 10.3390/cancers14102457 (PMC9139650; doi:10.3390/cancers14102457)
Supplement: Supplementary file 1 [file cancers-14-02457-s001.zip › cancers-1701391-supplementary.pdf]

Table S1. Reproducibility and accuracy of variant detection in the HD753 reference sample analyzed in 10 different runs (R4-R13). The variants in genes are given as the abbreviated change of amino acids. Variant allele frequencies (VAF) in percentages are provided for each run as well as the mean VAF and the standard deviation (SD). The last column provides the biological class of each variant according to the ComPerMed guidelines. Only the VAFs of the Pathogenic variants are provided by HorizonDx (HDx).

													**TSO500 VAF in 10 runs**		
**Gene**	**Change**	**HDx VAF (%)**	**VAF R04**	**VAF R05**	**VAF R06**	**VAF R07**	**VAF R08**	**VAF R09**	**VAF R10**	**VAF R11**	**VAF R12**	**VAF R13**	**mean (%)**	***SD***	**Biological Class**
BRAF	V600E	18,2	17,7	16,3	16,0	12,3	15,3	14,2	13,1	19,1	16,2	19,3	15,9	*2,3*	Pathogenic
PIK3CA	H1047R	16,7	18,0	20,5	18,1	18,4	22,0	18,1	14,2	17,7	15,5	15,3	17,8	*2,4*	Pathogenic
BRCA2	K1691Nfs*	5,6	4,4	7,8	4,0	7,7	6,5	9,2	2,7	4,7	7,7	6,3	6,1	*2,1*	Pathogenic
EGFR	A767_V769dup	5,6	3,8	3,1	2,5	2,3	3,7	3,7	4,4	2,6	3,0	2,5	3,2	*0,7*	Pathogenic
GNA11	Q209L	5,6	5,3	3,8	7,3	4,9	3,3	5,5	5,9	4,2	4,5	4,2	4,9	*1,2*	Pathogenic
KRAS	G13D	5,6	3,8	2,9	6,2	3,7	5,0	5,6	6,8	4,8	2,9	6,5	4,8	*1,5*	Pathogenic
PIK3CA	E545K	5,6	3,9	4,0	2,4	6,2	5,3	5,0	5,0	7,9	5,4	3,5	4,9	*1,5*	Pathogenic
EGFR	E746_A750del	5,3	5,8	4,0	4,4	3,3	4,4	3,5	7,4	2,8	5,0	4,9	4,6	*1,3*	Pathogenic
EGFR	G719S	5,3	3,9	9,6	5,2	2,4	6,7	3,9	5,2	4,2	4,7	5,9	5,2	*2,0*	Pathogenic
AKT1	E17K	5,0	2,9	3,9	3,5	2,9	4,5	2,1	3,4	3,5	4,2	2,8	3,4	*0,7*	Pathogenic
RNF43	G659fs*	np	22,6	21,2	19,1	22,6	27,3	20,0	21,0	19,0	18,4	22,1	21,3	*2,6*	Likely Pathogenic
TP53	E171*	np	13,4	9,7	14,4	11,5	11,3	12,2	13,3	11,6	11,6	13,4	12,2	*1,4*	Likely Pathogenic
NF2	P275fs*	np	12,4	8,2	11,4	10,3	12,3	12,8	11,2	8,6	10,4	11,3	10,9	*1,5*	Likely Pathogenic
ARID1A	D1850fs*	np	8,7	12,1	11,6	10,8	11,0	9,4	11,6	10,2	11,5	8,8	10,6	*1,2*	Likely Pathogenic
HNF1A	P379fs*	np	9,1	11,4	11,2	11,0	6,5	10,1	9,8	14,0	9,0	12,9	10,5	*2,1*	Likely Pathogenic
NCOR1	Q1833fs*	np	9,6	6,0	12,6	13,1	14,1	10,2	9,7	9,8	8,9	9,3	10,3	*2,4*	Likely Pathogenic
ARID1A	P1115fs*	np	9,5	10,6	8,7	10,8	9,4	11,4	8,9	11,2	7,5	8,2	9,6	*1,3*	Likely Pathogenic
CDKN2A	R58*	np	10,6	10,1	11,3	8,0	5,6	12,4	9,8	7,9	9,7	9,2	9,5	*1,9*	Likely Pathogenic
NF1	N2341fs*	np	10,9	6,9	10,0	5,6	13,0	8,9	11,0	9,1	9,7	7,9	9,3	*2,1*	Likely Pathogenic
NF1	Y628fs*	np	6,4	7,0	12,4	11,0	12,2	8,9	10,0	6,6	8,6	9,7	9,3	*2,2*	Likely Pathogenic
KMT2A	P773fs*	np	7,0	10,0	8,3	8,1	10,2	10,5	8,7	9,2	9,6	7,1	8,9	*1,2*	Likely Pathogenic
FANCA	E345fs*	np	10,4	8,6	8,3	8,1	7,5	8,2	10,8	6,8	8,7	10,0	8,7	*1,3*	Likely Pathogenic
TET2	S268*	np	10,5	5,0	7,5	11,9	4,4	6,2	6,3	10,4	7,1	7,5	7,7	*2,5*	Likely Pathogenic
STK11	F354L	np	6,4	6,4	6,5	10,7	6,9	7,7	7,3	7,0	5,7	8,1	7,3	*1,4*	Likely Pathogenic
BCOR	Q1208fs*	np	10,6	4,4	6,1	4,8	6,8	6,5	6,1	6,8	9,7	7,0	6,9	*1,9*	Likely Pathogenic
CDKN2A	R24fs*	np	9,4	7,0	5,5	7,8	3,1	5,9	11,0	9,2	4,9	4,9	6,9	*2,5*	Likely Pathogenic
TP53	S241C	np	6,6	6,1	6,9	7,5	6,6	4,6	5,5	9,2	6,3	6,2	6,5	*1,2*	Likely Pathogenic
TP53	S241F	np	5,5	6,1	6,9	7,5	6,6	4,6	5,5	9,2	6,3	6,2	6,4	*1,3*	Likely Pathogenic
CTNNB1	S45del	np	5,5	5,3	5,4	6,1	6,3	4,7	5,6	4,7	4,9	6,7	5,5	*0,7*	Likely Pathogenic
FBXW7	S668fs*	np	4,6	6,5	4,2	6,2	5,0	5,1	5,3	4,4	4,3	4,9	5,0	*0,8*	Likely Pathogenic
HNF1A	P291fs*	np	3,7	3,3	3,5	4,4	5,9	2,6	3,4	3,4	4,1	3,4	3,8	*0,9*	Likely Pathogenic

*del: deletion; dup: duplication; fs: frameshift; *: termination; np: not provided*

Table S2 . Analytical sensitivity of SNVs and indels in the retrospective and prospective FFPE samples. All unique gene variants are listed with their HGVS annotation. The variant type (SNV or indel), variant allele frequency (VAF) and coverages (Cov) are provided. Variants with a wide range of VAF and coverage were included.

Gene	variant	variant type	VAF	Cov
BRAF	c.1397G>A;p.(Gly466Glu)	SNV	34%	214
BRAF	c.1406G>A;p.(Gly469Glu)	SNV	36%	141
BRAF	c.1781A>G;p.(Asp594Gly)	SNV	17%	378
BRAF	c.1799T>A; p.(Val600Glu)	SNV	41%	370
BRCA1	c.1630C>T; p.(Gln544*)	SNV	90%	252
BRCA1	c.2722G>T; p.(Glu908*)	SNV	46%	439
BRCA1	c.3331_3334delCAAG; p.(Gln1111Asnfs*5)	indel	58%	208
BRCA1	c.5341-6_5349del;p.(?)	indel	34%	217
BRCA2	c.4440T>G; p.(Tyr1480*)	SNV	97%	87
BRCA2	c.5286T>A; p.(p.Tyr1762*)	SNV	58%	72
BRCA2	c.5645C>A;p.(Ser1882*)	SNV	79%	42
CTNNB1	c.110C>A; p.(Ser37Tyr)	SNV	19%	588
EGFR	c.2237_2251del;p.(Glu746_Thr751delinsAla)	indel	55%	678
EGFR	c.2310_2311ins;p.(Asp770_Asn771insGlyPhe)	indel	44%	1050
EGFR	c.2369C>T;p.(Thr790Met)	SNV	85%	7884
EGFR	c.2573T>G;p.(Leu858Arg)	SNV	92%	7496
ESR1	c.1387T>C; p.(Ser463Pro)	SNV	23%	1030
ESR1	c.1607T>A;p.(Leu536His)	SNV	82%	39
FBXW7	c.1394G>A;p.(Arg465His)	SNV	10%	231
FGFR3	c.742C>T; p.(Arg248Cys)	SNV	11%	447
GNAS	c.2531G>A;p.(Arg844His)	SNV	11%	462
HRAS	c.34G>A;p.(Gly12Ser)	SNV	6%	437
HRAS	c.34G>T;p.(Gly12Cys)	SNV	35%	563
IDH1	c.395G>A;p.(Arg132His)	SNV	37%	59
KIT	c.1727T>C;p.(Leu576Pro)	SNV	41%	265
KRAS	c.34G>T;p.(Gly12Cys)	SNV	8%	172
KRAS	c.35G>A;p.(Gly12Asp)	SNV	49%	246
KRAS	c.35G>C; p.(Gly12Ala)	SNV	17%	356
KRAS	c.35G>T;p.(Gly12Val)	SNV	5%	56
KRAS	c.38G>A;p.(Gly13Asp)	SNV	6%	719
KRAS	c.182A>G;p.(Gln61Arg)	SNV	42%	95
KRAS	c.183A>C;p.(Gln61His)	SNV	31%	118
NRAS	c.38G>T;p.(Gly13Val)	SNV	12%	1071
NRAS	c.183A>C; p.(Gln61His)	SNV	29%	218
PDGFRA	c.1698_1712del;p.(Ser566_Glu571delinsArg)	indel	49%	904
PIK3CA	c.263G>A; p.(Arg88Gln)	SNV	29%	196
PIK3CA	c.1624G>A; p.(Glu542Lys)	SNV	23%	179
PIK3CA	c.1633G>A;p.(Glu545Lys)	SNV	22%	168
PIK3CA	c.1634A>G;p.(Glu545Gly)	SNV	13%	149
PIK3CA	c.3140A>G;p.(His1047Arg)	SNV	38%	26
POLE	c.847dupC; p.(Leu283Profs*5)	indel	8%	462
POLE	c.857C>G; p.(Pro286Arg)	SNV	20%	588
POLE	c.1231G>T; p.(Val411Leu)	SNV	35%	694
SMAD4	c.1081C>T;p.(Arg361Cys)	SNV	24%	525
SMAD4	c.1082G>A;p.(Arg361His)	SNV	14%	157
TERT	c.-124C>T (C228T)	SNV	20%	149
TERT	c.-146C>T (C250T)	SNV	31%	212

del: deletion; fs: frameshift; ins: insertion; *: termination

Table S3 . Analytical sensitivity and specificity of exon skipping and fusion gene detection analysed on RNA from 25 positive retrospective and 54 random prospective samples. The confirmed fusion events are listed in the left columns while the 5 structural variants in prospective samples that could not be checked due to the absence of an orthogonal assay are shown in the right columns.

Confirmed structural variant	supp reads
EGFR vIII	2144
MET ex14	6885
CCDC6::RET	4550
CD74::ROS1	250
EML4::ALK var1	162
EML4::ALK var3a	24
ETV6::NTRK3	1170
EWSR1::ERG	3376
EWSR1::FLI1	1963
EWSR1::NFATC2	7287
FGFR3::BAIAP2L1	1474
FGFR3::TACC3	140
HIP1::ALK	460
KIF5B::RET	795
LMNA::NTRK1	2082
NCOA4::RET	1992
NPM1::ALK	40
PAX3::FOXO1	5593
PAX8::PPARG	1419
SDC4::ROS1	2162
SLC34A2::ROS1	272
SLC45A3::BRAF	1559
TMPRSS2::ERG	1804
TPM3::NTRK1	565

Structural variant not confirmed	supp reads	comment
ERG::TTC3	11	del(21) unknown
BRCA2::SLC7A1	392	del(13) unknown
SLC45A3::ETV1	1982	t(1;7) known
GLCCI1::EML4	120	t(2;7) unknown
NECTIN2::ALK	16	t(2;19) unknown

Table S4. Limit-of-Detection for SNVs (16) and indel (8) detection. The samples were diluted to the indicated percentages (contribution to the mixed sample) in a second sample.

		contribution to the mixed sample			
**Gene**	**variant**	**15%**	**50%**	**85%**	**100%**
EGFR	T790M	nt	17%	19%	19%
BRAF	V600E	1%	6%	31%	75%
PIK3CA	H1047R	3%	5%	26%	38%
PIK3CA	N345K	8%	31%	48%	70%
TP53	R175K	2%	11%	31%	87%
BRAF	V600E	4%	13%	24%	23%
PIK3CA	H1047R	2%	7%	10%	11%
GNAS	R844H	1%	5%	7%	9%
EGFR	L858R	47%	79%	90%	92%
EGFR	T790M	44%	73%	83%	85%

		contribution to the mixed sample			
**Gene**	**variant**	**25%**	**50%**	**75%**	**100%**
PTEN	R233*	1%	1%	5%	nt
FBXW7	R505H	12%	15%	21%	nt
TET2	Q705*	37%	57%	61%	nt
KRAS	G12V	5%	5%	nt	nt
GNAS	R844H	4%	8%	nt	nt
PTEN	Q245*	10%	13%	17%	nt
BRCA1	Q1111Nfs*	nt	18%	31%	58%
EGFR	ex19del15bp	24%	36%	42%	nt
BRCA2	Q1782Tfs*	6%	4%	7%	13%
TET2	Q1861Efs*	2%	6%	8%	15%
POLE	L283Pfs*	3%	4%	4%	8%
ARID1A	G1850Tfs*	1%	3%	6%	9%
PTEN	L267Rfs*	2%	1%	14%	nt
APC	T1556Nfs*	2%	5%	10%	nt

del: deletion; fs: frameshift; nt: not tested

Table S5. Limit of Detection for amplifications. Fold changes (FC) obtained for the genes with a known copy number variation (CNV) in retrospective samples analysed with the AmpliSeq assay on the original sample, and with the TSO500 assay on different contributions of that sample to the mixtures. FC's below the set threshold of 1.8 are indicated in italic.

				Contribution to the mixture		
Sample	TC	CNV	AmpliSeq	75%	50%	25%
A1	50%	EGFR	13.0	12.0	8.9	5.3
A2	40%	ERBB2	5.1	4.7	3.2	2.0
		CCND1	np	2.8	2.1	*1.4*
		FGF19	np	3.2	2.3	*1.6*
		FGF4	np	3.1	2.2	*1.6*
		FGF3	np	3.0	2.1	*1.5*
A3	30%	EGFR	2.0	2.0	*1.7*	*1.3*
A4	60%	none	nd	nd	nd	nd

nd: not detected; np: not present

Table S6. Limit of Detection of fusion gene analysis in 4 FFPE samples demonstrating that a 4-fold dilution (25% contribution) was still able to detect the fusion event.

			Contribution to the mixture		
Sample	TC	fusion	100%	75%	25%
F1	70%	KIF5B::RET	2878	1398	322
F2	35%	EML4::ALK	427	520	82
F3	90%	SLC45A2::ERG	126	67	16
F4	50%	none	0	0	0

Table S7. Limit of detection of the SeraSeq RNA reference sample undiluted, and diluted 5- and 15-times. The number of supporting reads is indicated for both exon splicing and gene fusion events.

fusion	*undiluted*	*5x diluted*	*15x diluted*
EGFR vIII	61	22	12
MET ex14 splice	32	*nd*	*nd*
SLC45A3-BRAF	54	14	*nd*
FGFR3-TACC3	174	39	16
FGFR3-BAIAP2L1	79	27	8
KIF5B-RET	94	19	8
NCOA4-RET	118	19	7
TMPRSS2-ERG	65	18	13
EML4-ALK	70	8	5
CD74-ROS1	8	*nd*	*nd*
ETV6-NTRK3	67	6	8
LMNA-NTRK1	71	20	8
TPM3-NRTK1	93	8	*nd*
PAX8-PPARG	56	10	7
SLC34A2-ROS1	18	*nd*	*nd*
EGFR-SEPT14	64	13	*nd*

nd: not detected

Table S8. Accuracy of the TSO500 analysis on SeraSeq FFPE Tumor Fusion RNA Reference material v2. Both exon skipping events and gene fusions are listed as well as the number of supporting fusion reads (# supp reads) obtained by TSO500 analysis for each rearrangement.

Rearrangement	# supp reads
EGFR vIII	61
MET ex14 splice	32
SLC45A3-BRAF	54
FGFR3-TACC3	174
FGFR3-BAIAP2L1	79
KIF5B-RET	94
NCOA4-RET	118
TMPRSS2-ERG	65
EML4-ALK	70
CD74-ROS1	8
ETV6-NTRK3	67
LMNA-NTRK1	71
TPM3-NRTK1	93
PAX8-PPARG	56
SLC34A2-ROS1	18
EGFR-SEPT14	64
